# Latent class growth modeling of depression and anxiety in older adults: an 8-year follow-up of a population-based study

**DOI:** 10.1186/s12877-021-02501-6

**Published:** 2021-10-13

**Authors:** Yanzhao Cheng, Lilian Thorpe, Rasel Kabir, Hyun Ja Lim

**Affiliations:** 1 Collaborative Biostatistics Program, School of Public Health, University of Saskatchewan, Saskatoon, Canada; 2 Department of Community Health and Epidemiology, College of Medicine, University of Saskatchewan, 104 Clinic Place, Saskatoon, SK S7N2Z4 Canada

**Keywords:** Depressive disorder, Trajectory, Anxiety, Longitudinal studies, Older adults

## Abstract

**Background:**

Depression and anxiety are common mental health conditions in the older adult population. Understanding the trajectories of these will help implement treatments and interventions.

**Aims:**

This study aims to identify depression and anxiety trajectories in older adults, evaluate the interrelationship of these conditions, and recognize trajectory-predicting characteristics.

**Methods:**

Group-based dual trajectory modeling (GBDTM) was applied to the data of 3983 individuals, aged 65 years or older who participated in the Korean Health Panel Study between 2008 and 2015. Logistic regression was used to identify the association between characteristics and trajectory groups.

**Results:**

Four trajectory groups from GBDTM were identified within both depression and anxiety outcomes. Depression outcome fell into “low-flat (87.0%)”, “low-to-middle (8.8%)”, “low-to-high (1.3%)” and “high-stable (2.8%)” trajectory groups. Anxiety outcome fell into “low-flat (92.5%)”, “low-to-middle (4.7%)”, “high-to-low (2.2%)” and “high-curve (0.6%)” trajectory groups. Interrelationships between depression and anxiety were identified. Members of the high-stable depression group were more likely to have “high-to-low” or “high-curved” anxiety trajectories. Female sex, the presence of more than three chronic diseases, and being engaged in income-generating activity were significant predictors for depression and anxiety.

**Conclusions:**

Dual trajectory analysis of depression and anxiety in older adults shows that when one condition is present, the probability of the other is increased. Sex, having more than three chronic diseases, and not being involved in income-generating activity might *increase risks* for both depression and anxiety. Health policy decision-makers may use our findings to develop strategies for preventing both depression and anxiety in older adults.

**Supplementary Information:**

The online version contains supplementary material available at 10.1186/s12877-021-02501-6.

## Introduction

As well as the increased physical comorbidities associated with aging, older adults are known to be at risk of both depression and anxiety disorders [1]. The research focused on studying the comorbidity of depression and anxiety in elders has increased recently [2]. One likely reason for this is the aging population, with the proportion of older adults increasing every year in almost every country [3]. In South Korea, concerns related to aging are substantial, as individuals 65 years or older constitute approximately 14% of the overall population, which is more than twice the proportion of people aged 14 or younger [4]. Another reason for the combined interest in depression and anxiety is that a variety of physical health conditions common to aging may increase the development of mental health problems in the older adult population. For example, older adults who suffer one or more chronic diseases have a greater chance of developing late-onset depression and/or anxiety [5–7].

Although it has been suggested that comorbidity between depression and anxiety is less in the older than younger adults [8], it is still of considerable concern in older adults. Beekman et al. found that 47.5% of individuals aged 65 years or older who suffer from depression also have a comorbid anxiety disorder [9]. Other research from Gould based on the Health and Retirement Study [10] to found that, compared with elevated depressive symptoms, anxiety was associated with greater multimorbidity in older adults. Many studies showed that older adults with multimorbidity had more risk to be depressed or anxious compared to those without multimorbidity [10–12]. Thus, it was important to consider the comorbidity of depression and anxiety.

A Korean study including 1204 older adults showed that 22.8% had comorbid anxiety and depression [13]. In terms of psychopathology, older adults with both anxiety and depression have been found to have greater severity than patients with only one disorder [1]. Depression comorbid with anxiety has resulted in more severe somatic symptoms than patients with only depression [14, 15]. Moreover, poorer social function and higher suicide rates are more likely to persist in the comorbid depression and anxiety group than those with only depression or anxiety [16, 17].

Common risk factors for both depression and anxiety in older adults have been categorized into biological, psychological and social factors [18], including the influences of physical illness, disability, bereavement, and others. Nevertheless, longitudinal studies suggest that there are some differences in risk factors between late-life depression and anxiety. Depression and anxiety have both been associated with cognitive impairments. Multiple studies have shown that depressive disorder was associated with cognitive impairment such as deficits in verbal and nonverbal learning, memory, attention, visual and auditory processing, everyday problem-solving ability directly and indirectly, executive function, processing speed, and reasoning [19–21]. Anxious subjects did not differ significantly from depressed subjects in any measure of cognitive function [22]. However, anxiety was more often associated with short-term and delayed memory, blackouts/memory loss, complex visuospatial performance and visual learning, poorer performance on verbal working memory, poor global cognitive functioning, working memory, inhibition, information processing speed, problem-solving including concept formation and mental flexibility [22, 23].

Interestingly, psychological risk factors have been found to be similar for depression and anxiety [9, 24, 25]. Social risk factors such as marital status and social network size have generally correlated with depression [26–28]. Social risk factors are risks related to social support or social ties/isolation, such as one’s social network and frequency of contact with relatives and friends [29]. In contrast, risk factors associated with anxiety but not depression include being childless, having experienced traumatic life events, and having low income [9, 24, 25, 30–34].

Previously reported trajectory analyses of both depression and anxiety have usually been developed based on data from children and adolescents [35–38]. To study similar trajectories in older adults, researchers have generally focused only on depression [39–44]. However, depression and anxiety trajectories have rarely been studied simultaneously. Three studies were found exploring the development of depression and anxiety trajectories in older adults [45–47]. Holmes et al. [45] and Spinhoven et al. [47] used latent growth mixture modeling to identify different course trajectories, Rzewuska et al. [46] used latent class analysis to explore depression and anxiety trajectories separately. The present study seeks to examine the longitudinal association between depression and anxiety in the older population using a joint trajectory modeling method. The aims of our study were (i) to identify distinct trajectories of depression or anxiety; (ii) to describe the baseline characteristics among trajectory groups of depression or anxiety; (iii) to estimate conditional probabilities of anxiety given depression and conditional probabilities of depression given anxiety; (iv) to evaluate the strength of association between predictors of depression as well as predictors of anxiety.

## Methods

### Data and sample

This study utilized a subset of a longitudinal survey called the Korea Health Panel Study (KHPS), which is an official database designed and maintained since 2008 by the Korea Institute for Health and Social Affairs and the National Health Insurance Service.

More detailed descriptions of the study design and profile have been published elsewhere [48]**.** Briefly, KHPS used a stratified sampling frame taken from the Korean Population and Housing Census in 2005. Sample weights for the KHPS were calculated after adjusting for unequal selection probabilities/non-responses and making a population distribution disclosure via post-stratification corresponding to the sample distribution.

The KHPS began in 2008 and incorporated a total of 24,616 participants from 7387 households. Due to ongoing dropouts and to secure statistical reliability, new household members were added. In 2014, KHPS was strengthened with the addition of more the 2520 households to mitigate attrition. Using computer-assisted personal interviews, trained staff collected the data at three general levels: household, individual, and case-based. Comprehensive assessments of the use of healthcare services, cost of healthcare, and potentially influencing these have been conducted annually since 2008. Further details of the KHPS administration and mission are available on the KHPS website [49]. Overall, 3983 adults who were 65 or older were studied during the study period between 2008 and 2015. Additional subjects were added in the year 2014 to the original data collected in the year 2008. The follow-up participation rates among this study’s sample were 91.0, 63.4, 60.6, 56.5, 53.4, 50.0 and 46.4% from year 2009 to 2015, respectively. The average number of responses was 5.2 times (Fig. S1 in Supplement Material).

### Measures

The data collection methods for the KHPS involved the investigators visiting the target households and using a computer-assisted personal interviewing (CAPI) technique.

Covariates included sex, age, education, marital status, residential area, number of members in the household, household composition type, housing type, current chronic disease status, private health insurance, household income quantile, and household expense [48]. Age was categorized as 65–69, 70–74, 75–79, and 80 years and older. Sex was coded 0 = male and 1 = female. Education was coded as 0 = no education, 1 = Grade 1–6, and 2 = Grade 7 or higher. Residential area was categorized into two areas and coded as metro city = 0 and non-metro city = 1. Household composition type was categorized as 1 = living alone, 2 = living with a spouse, and 3 = other mixed living arrangements. Housing type was categorized as 1 = detached house, 2 = apartment, and 3 = other types of houses. Exercise and walking were scored separately on an 8-point Likert scale that asked respondents how many days during the past week you did intensive/moderate physical activity or walked more than 10 min a day. Responses ranged from 0 to 7 (none = 0, once a week = 1, 2 days a week = 2, 3 days a week = 3, 4 days a week = 4, 5 days a week = 5, 6 days a week = 6, 7 days a week = 7). Drinking was scored on an 8-point Likert scale that asked, “Over the past year, how often did you drink alcohol?” Again an 8-point Likert scale (never = 0; recently non-drink = 1, less than once per month = 2, once per month = 3, 2–3 times per month = 4; once per week = 4; 2–3 times a week = 6; almost daily = 7). In our study, exercise and walking variables were categorized as ‘none’, ‘≤ 3 days/week’ and ‘> 3 days/week’. Drinking variable was categorized as ‘none’, ‘less than twice/week’, ‘2–4 times/week’ and ‘almost daily’. In the study database, chronic diseases were hypertension, heart disease, diabetes, back pain, cataracts, osteoporosis, and arthritis, loss of hearing or vision which was common in the older adults and lead to impaired daily functioning. The number of current chronic diseases was coded as ‘yes’ = having 3 or more of these chronic diseases, and ‘no’ = otherwise.

Diagnostic criteria for depression and anxiety disorder were based on DSM-5 (The Diagnostic and Statistical Manual of Mental Disorders, Fifth Edition). The main dichotomous outcomes of depression and anxiety were collected from medical administrative expenditure data including prescription drug receipts or medical institutions/pharmacies. If there was any use of inpatient treatment, outpatient treatment, or emergency-service utilization each year from the case-based survey relevant to depression or anxiety, the outcome was assigned a value of 1, otherwise, the outcome was assigned a value of 0.

### Statistical analysis

The analysis commenced with basic descriptive statistics to characterize the study sample.

General group-based trajectory modeling (GBTM) with single repeated measurement outcome, also called latent class growth modeling, can identify unobserved heterogeneous subgroups within the sample population and develop trajectories based on the trends within them [50]. Individuals are placed into the most likely subgroups relying on the largest posterior probability (i.e., the probability that an individual with a particular outcome pattern belongs to a particular group among those suggested by the model) [51].

Group-based dual trajectory modeling (GBDTM) extended from GBTM is a joint model to determines the trajectories of two associated outcomes based on the conditional probability of developing a given outcome, such as depression and anxiety in our study [52]. The relationship between the two outcomes has been labeled as “comorbidity” or “heterotypic continuity” [53]. “Comorbidity” recognizes multiple illness states occurring at the same time [54]. “Heterotypic continuity” recognizes that two outcomes may be linked within an individual but do not occur simultaneously, introducing the possibility of causality [55]. In GBDTM, the linkage of trajectory groups identified within the respective two associated outcomes relies on the conditional probabilities. For example, since depression and anxiety are diagnosed as co-current events in our study, the conditional probabilities for both depression given anxiety and anxiety given depression were considered [56]. Therefore, this conditional probability represents the likelihood of a person having depression if anxiety is present and vice versa [53].

To select the most appropriate number of trajectories, separate GBTM for depression and anxiety with the number of trajectory groups from two to five were tried. All the trajectories from the model were assumed to be linear. Bayesian Information Criterion (BIC) [57], Akaike Information Criterion (AIC) [58], and the average posterior probability [59] were utilized to evaluate the model fit. Initially, the separate GBTM for depression and anxiety were conducted. Then, based on the result from the separate GBTM, GBDTM was developed. The missing values of response variable can be imputed missing at random (MAR) assumption. i.e., when data are MAR, information from the dataset can be used to impute missing data prior to input into the trajectory model [60].

SAS programming 9.4 and Proc Traj package was used to fit trajectory modeling, which employs an imputation technique to assign values for missing data. The significant level of this study was set at α = 0.05.

## Results

3983 participants, aged 65 or older from KHPS, were included during the study period between 2008 and 2015. In this sample, 57% were female, and the average age at baseline measurement was 72.4 (SD ± 6) years. Among these participants, 63% had never received any education or only finished elementary school, 62.5% lived with their spouse, and 1.6% lived alone. The majority (83%) reported that their income level was lower than the median income level of the complete survey sample, and 37% were still involved in income-generating activities. Only 38.2% lived in metro-cities, and 57.4% resided in a detached house.

**Fig. 1:**
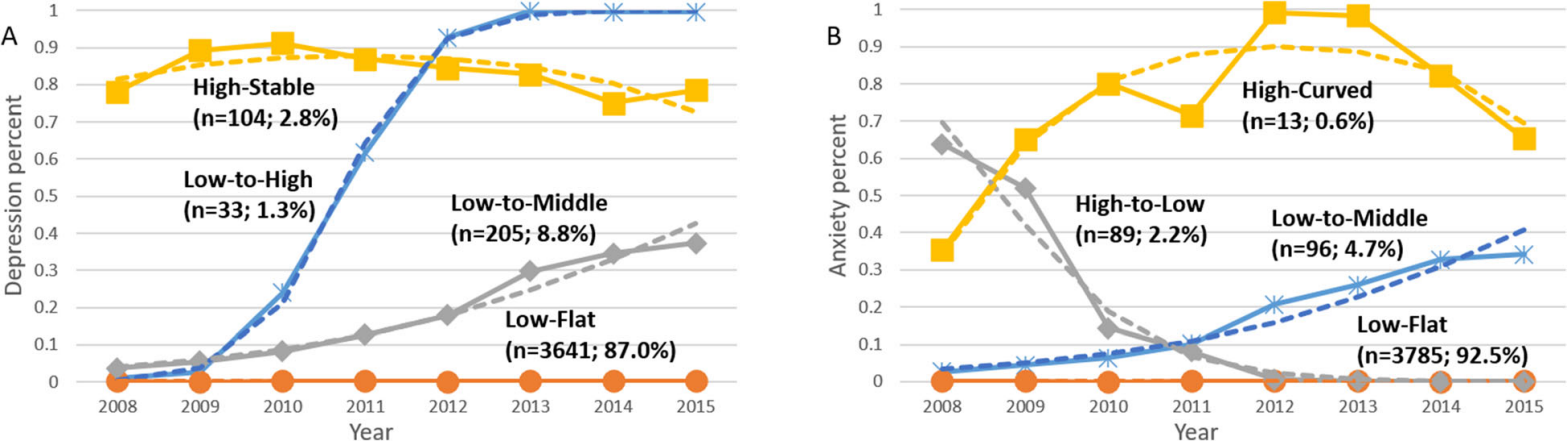
Depression and Anxiety Trajectories. The solid line indicates the observed value; the dot line the dashed line indicates the predicted value. A and B are depression and anxiety trajectories in group-based dual trajectory modeling, respectively

Based on model selection criteria, four trajectory groups were identified as the best fit for both the depression and anxiety outcomes from GBDTM (Table 1). Figure 1 presents trajectory groups with depression and anxiety. Within the depression outcome, four distinct trajectory groups were identified using dual trajectory modeling (Fig. 1A): low-flat trajectory group (87.0%), low-to-middle trajectory group (8.8%), low-to-high (1.3%) and high-stable trajectory group (2.8%). Likewise, within anxiety, four distinct trajectory groups were also identified using dual trajectory modeling (Fig. 1B): low-flat trajectory group (92.5%), low-to-middle trajectory group (4.7%), high-to-low trajectory group (2.2%) and high-curved trajectory group (0.6%).

**Table 1:** Goodness of fit to select the optimal number of trajectory group for depression and anxiety

Number of trajectories	Depression			Anxiety		
	BIC	AIC	PP	BIC	AIC	PP
**2**	− 2534.8	− 2530.7	0	− 1525.8	− 1521.7	0
**3**	− 2403.8	− 2397.2	0	− 1484.2	− 1477.6	0
**4**	− 2363.7	− 2354.7	0.83	− 1476.3	− 1467.2	1
**5**	− 2365.3	− 2353.8	0.17	− 1489.8	− 1478.3	0

*BIC* Bayesian information criterion, *AIC* Akaike information criterion, *PP* Posterior probability

The subpopulation characteristics are described based on the respective trajectory groups from GBDTM (Tables 2 and 3).

**Table 2:** Distribution of Baseline Characteristics by Depression Trajectory Groups (n, %)

	Trajectory groups				***P***-value
	Low-flat ***N*** = 3641	Low-to-middle ***N*** = 205	Low-to-high ***N*** = 33	High-stable ***N*** = 104	
**Anxiety**					
**Low-flat**	3519 (96.7)	162 (79.0)	28 (84.9)	76 (73.1)	< 0.0001
**Low-to-middle**	61 (1.7)	29 (14.2)	3 (9.1)	3 (2.9)	
**High-to-low**	57 (1.6)	7 (3.4)	2 (6.1)	23 (22.1)	
**High-curved**	4 (0.11)	7 (3.4)	0 (0.0)	2 (1.9)	
**Sex**					
**Male**	1619 (44.5)	59 (28.8)	9 (27.3)	27 (26.0)	< 0.0001
**Female**	2022 (55.5)	146 (71.2)	24 (72.7)	77 (74.0)	
**Age**					
**65–69**	1405 (38.6)	86 (42.0)	11 (33.3)	32 (30.8)	0.009
**70–74**	1099 (30.2)	73 (35.6)	11 (33.3)	32 (30.8)	
**75–79**	666 (18.3)	37 (18.1)	8 (24.2)	29 (27.9)	
**≥ 80**	471 (12.9)	9 (4.4)	3 (9.1)	11 (10.6)	
**Marital status**					
**Married**	1272 (35.0)	70 (34.2)	12 (36.4)	36 (34.6)	0.993
**Single/divorced/widowed**	2368 (65.0)	135 (65.8)	21 (63.6)	68 (65.4)	
**Education**					
**None**	728 (20.0)	39 (19.0)	8 (24.2)	24 (23.1)	0.308
**Elementary**	1551 (42.6)	101 (49.3)	13 (39.4)	40 (38.5)	
**Middle/High**	1079 (29.6)	55 (26.8)	10 (30.3)	37 (35.6)	
**University**	283 (7.8)	10 (4.9)	2 (6.1)	3 (2.9)	
**Smoking**					
**No**	1982 (58.9)	143 (69.8)	22 (66.7)	71 (74.0)	0.0006
**Previous**	442 (13.1)	12 (5.9)	2 (6.1)	11 (11.5)	
**Current**	944 (28.0)	50 (24.4)	9 (27.3)	14 (14.6)	
**Drinking**					
**No**	1323 (39.3)	101 (49.3)	19 (57.6)	52 (54.2)	0.002
**Less than twice per week**	1507 (44.7)	84 (41.0)	12 (36.4)	37 (38.5)	
**2–3 times per week**	266 (7.9)	10 (4.9)	0 (0.0)	2 (2.1)	
**Almost daily**	274 (8.1)	10 (4.9)	2 (6.1)	5 (5.2)	
**Residential area**					
**Metro-city**	1392 (38.2)	75 (36.6)	14 (42.4)	39 (37.5)	0.920
**Not Metro-city**	2249 (61.8)	130 (63.4)	19 (57.6)	65 (62.5)	
**Housing type**					
**Detached House**	2094 (57.5)	119 (58.1)	17 (51.5)	56 (53.9)	0.770
**Apartment**	512 (14.1)	22 (10.7)	5 (15.2)	17 (16.4)	
**Others**	1035 (28.4)	64 (31.2)	11 (33.3)	31 (29.8)	
**Disability**					
**No**	2931 (80.5)	161 (78.5)	26 (79.8)	73 (70.2)	0.068
**Yes**	710 (19.5)	44 (21.5)	7 (21.2)	31 (29.8)	
**Home ownership**					
**Own**	2790 (76.6)	159 (77.6)	27 (81.8)	66 (63.5)	0.015
**Lease**	851 (23.4)	46 (22.4)	6 (18.2)	38 (36.5)	
**Living arrangement**					
**Alone**	59 (1.48)	1 (0.5)	0 (0.0)	2 (1.9)	0.137
**Couple only**	2231 (61.3)	145 (70.7)	21 (63.6)	69 (66.4)	
**Others**	1351 (37.1)	59 (28.8)	12 (36.4)	33 (31.7)	
**Walking**					
**None**	625 (18.6)	33 (16.1)	5 (15.2)	27 (28.1)	0.044
**≤ 3 days/week**	417 (12.4)	34 (16.6)	6 (18.2)	16 (16.7)	
**> 3 days/week**	2328 (69.1)	138 (67.3)	22 (66.7)	53 (55.2)	
**Medium/Intensive**					
**Physical activity**					
**None**	2247 (66.7)	134 (65.4)	27 (81.8)	74 (77.1)	0.181
**≤ 3 days/week**	326 (9.7)	21 (10.2)	3 (9.1)	7 (7.3)	
**> 3 days/week**	797 (23.7)	50 (24.4)	3 (9.1)	15 (15.6)	
**More than 3 chronic diseases** ^a^					
**Yes**	3175 (87.2)	198 (96.6)	32 (97.0)	102 (98.1)	< 0.0001
**No**	466 (12.8)	7 (3.4)	1 (3.0)	2 (1.9)	
**Economic Activity**					
**No**	2278 (62.6)	135 (65.9)	27 (81.8)	82 (78.9)	0.0007
**Yes**	1363 (37.4)	70 (34.1)	6 (18.2)	22 (21.1)	
**Income quantile, percentile**					
**< 20th**	1164 (44.1)	91 (50.8)	15 (45.5)	39 (55.7)	0.100
**20 – 40th**	603 (22.8)	39 (21.8)	9 (27.3)	15 (21.4)	
**40 – 60th**	425 (16.1)	32 (17.9)	6 (18.2)	8 (11.4)	
**60 – 80th**	238 (9.0)	12 (6.7)	3 (9.1)	7 (10.0)	
**80 – 100th**	210 (8.0)	5 (2.8)	0 (0.0)	1 (1.4)	

^a^Chronic diseases = hypertension, heart disease, diabetes, back pain, cataracts, osteoporosis, and arthritis, loss of hearing or vision

**Table 3:** Distribution of Baseline Characteristics by Anxiety Trajectory Groups (n, %)

	Trajectory groups				***P***-value
	Low-flat ***N*** = 3785	Low-to-middle ***N*** = 96	High-to-low ***N*** = 89	High-curved ***N*** = 13	
**Sex**					
**Male**	1664 (44.0)	26 (27.1)	20 (22.5)	4 (30.8)	< 0.0001
**Female**	2121 (56.0)	70 (72.9)	69 (77.5)	9 (69.2)	
**Age, years**					
**65–69**	1454 (38.4)	39 (40.6)	37 (41.6)	4 (30.8)	0.995
**70–74**	1156 (30.5)	29 (30.2)	26 (29.2)	4 (30.8)	
**75–79**	702 (18.6)	19 (19.8)	16 (18.0)	3 (23.1)	
**≥ 80**	473 (12.5)	9 (9.4)	10 (11.2)	2 (15.4)	
**Marital status**					
**Married**	2475 (65.4)	59 (61.5)	49 (55.1)	9 (69.2)	0.190
**Single/divorced/widowed**	1309 (34.6)	37 (38.5)	40 (44.9)	4 (30.8)	
**Education**					
**None**	753 (19.9)	20 (20.8)	24 (27.0)	2 (15.4)	0.194
**Elementary**	1611 (42.6)	50 (52.1)	35 (39.3)	9 (69.2)	
**Middle/High**	1133 (29.9)	22 (22.9)	25 (28.1)	1 (7.7)	
**University**	288 (7.6)	4 (4.2)	5 (5.6)	1 (7.7)	
**Smoking**					
**No**	2083 (59.4)	65 (68.4)	62 (72.1)	8 (61.5)	0.042
**Previous**	451 (12.9)	6 (6.3)	10 (11.6)	0 (0)	
**Current**	974 (27.8)	24 (25.3)	14 (16.3)	5 (38.5)	
**Drinking**					
**No**	1408 (40.1)	37 (39.0)	44 (51.2)	6 (46.2)	0.433
**Less than twice per week**	1554 (44.3)	45 (47.4)	35 (40.7)	6 (46.2)	
**2–3 times per week**	269 (7.7)	4 (4.2)	4 (4.7)	1 (7.7)	
**Almost daily**	279 (8.0)	9 (9.5)	3 (3.5)	0 (0.0)	
**Residential area**					
**Metro-city**	1392 (38.2)	14 (42.4)	75 (36.6)	39 (37.5)	0.920
**Not Metro-city**	2249 (61.8)	19 (57.6)	130 (63.4)	65 (62.5)	
**Housing type**					
**Detached House**	2173 (57.4)	59 (61.5)	47 (52.8)	7 (53.9)	0.811
**Apartment**	528 (14.0)	11 (11.5)	16 (18.0)	1 (7.7)	
**Others**	1084 (28.6)	26 (27.1)	26 (29.2)	5 (38.5)	
**Disability**					
**No**	3034 (80.2)	79 (82.3)	65 (73.0)	13 (100.0)	0.097
**Yes**	751 (19.8)	17 (17.7)	24 (27.0)	0 (0.0)	
**Home ownership**					
**Own**	2898 (76.6)	78 (81.2)	59 (66.3)	7 (53.9)	0.019
**Lease**	887 (23.4)	18 (18.8)	30 (33.7)	6 (46.2)	
**Living arrangement**					
**Alone**	61 (1.6)	1 (1.04)	0 (0)	0 (0)	0.631
**Couple only**	2336 (61.7)	67 (69.8)	55 (61.8)	8 (61.5)	
**Others**	1388 (36.7)	28 (29.2)	34 (38.2)	5 (38.5)	
**Walking**					
**None**	650 (18.5)	15 (15.8)	22 (25.6)	3 (23.1)	0.562
**≤ 3 days/week**	453 (12.9)	11 (11.6)	7 (8.1)	2 (15.4)	
**> 3 days/week**	2407 (68.6)	69 (72.6)	57 (66.3)	8 (61.5)	
**Medium/Intensive**					
**Physical activity**					
**None**	2349 (66.9)	59 (62.1)	63 (73.3)	11 (84.6)	0.493
**≤ 3 days/week**	340 (9.7)	9 (27)	8 (9.3)	0 (0.0)	
**> 3 days/week**	821 (23.4)	27 (28.4)	15 (17.4)	2 (15.4)	
**More than 3 chronic diseases** ^a^					
**Yes**	3319 (87.7)	88 (91.7)	87 (97.7)	0 (0.0)	0.001
**No**	466 (12.3)	8 (8.3)	2 (2.3)	13 (100.0)	
**Economic Activity**					
**No**	2382 (62.9)	61 (63.5)	70 (78.7)	9 (69.2)	0.024
**Yes**	1403 (37.1)	35 (36.5)	19 (21.4)	4 (30.8)	
**Income quantile, percentile**					
**< 20th**	1224 (44.4)	44 (51.2)	31 (44.9)	31 (44.9)	0.410
**20 – 40th**	628 (22.8)	19 (22.1)	18 (26.1)	18 (26.1)	
**40 – 60th**	446 (16.2)	11 (12.8)	13 (18.8)	13 (18.8)	
**60 – 80th**	246 (8.9)	9 (10.5)	5 (7.3)	5 (7.3)	
**80 – 100th**	210 (7.6)	3 (3.5)	2 (2.9)	2 (2.9)	

^a^Chronic diseases = hypertension, heart disease, diabetes, back pain, cataracts, osteoporosis, and arthritis, loss of hearing or vision

**Fig. 2:**
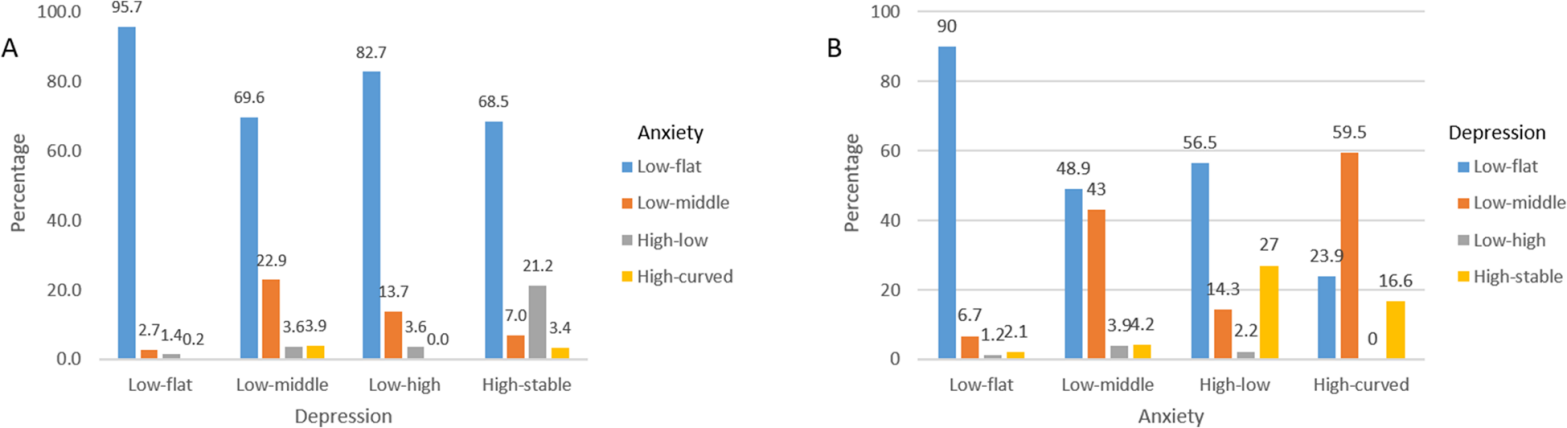
Conditional probability of anxiety given depression (**A**). Conditional probability of depression given anxiety (**B**). No covariate was adjusted

Conditional probabilities from GBDTM provided a clear view of the association between depression and anxiety in the dual trajectory model. The most likely anxiety trajectory regardless of depression trajectory was low-flat, with probabilities ranging from 95.7% probability in the low-flat depression group to 68.5% in the high-stable depression group (Fig. 2A). For interpretation of conditional probabilities from GBDTM in Fig. 2A, of subjects with low-flat depression (i.e., given a low-flat depression), 95.7% belong to the low-flat anxiety trajectory group. Likewise, 22.9% of low-to-middle depression trajectory participants were in the low-to-middle anxiety trajectory group, and 21.1% of the high-stable depression group followed a high-to-low anxiety trajectory. Persistent anxiety trajectories (high-curved) were only observed among respondents with low-to-middle or high-stable trajectory depression patterns and in small proportions (Fig. 2A). Figure 2B illustrates the conditional probability of depression given anxiety, suggesting that the highest depression trajectories (High-Stable depression) are seen in the highest anxiety groups (high-low and high-curved).

Univariate logistic regression analysis for depression and anxiety trajectory groups are presented in Tables S1 and S2 in Supplement Material. In multivariate analyses of depression (Table 4), compared to the low-flat depression group, individuals in the low-to-high depression trajectory were more likely to be in the low-to-middle anxiety trajectory group (OR = 5.80, 95% CI: 1.83–19.68, *p*-value = 0.005) and less likely to be participating in income-generating activity (OR = 2.79, 95% CI: 1.15–6.80, p-value = 0.024). Similarly, members of the low-to-middle depression group were also more likely to be in the low-to-middle anxiety trajectory group or the high-curved anxiety trajectory group, be female and have multiple chronic diseases. Participants in the high-stable depression trajectory group were more often in the low-to-middle or high-curved anxiety and were more likely to be female, have multiple chronic diseases, and not be income-generating activity compared to the low-flat depression group.

**Table 4:** Multivariate Logistic Regression Analyses with Depression Trajectory Groups. Estimation of odds ratio (OR) and 95% confidence interval (C.I.). Low-flat depression as the reference group

Variables	Low-to-middle		Low-to-high		High-stable	
	OR (95% CI)	***P***-value	OR (95% CI)	***P***-value	OR (95% CI)	***P***-value
**Anxiety**						
**Low-flat**	–		–		–	–
**Low-to-middle**	9.26 (5.63–15.22)	< 0.0001	5.80 (1.71–19.68)	0.005	2.67 (0.80–8.95)	0.111
**High-to-low**	2.19 (0.96–4.98)	0.061	3.64 (0.84–15.71)	0.084	10.65 (5.30–21.38)	< 0.0001
**High-curved**	25.91 (7.45–90.15)	< 0.0001	0.01 (0.01–99.9)	0.992	17.31 (3.04–98.68)	< 0.0001
**Sex**						
**Male**	–	–			–	–
**Female**	1.51 (1.07–2.12)	0.018			1.87 (1.02–3.41)	0.042
**More than 3 chronic diseases** ^a^						
**No**	–	–			–	–
**Yes**	3.96 (1.83–8.59)	0.0005			4.18 (1.01–17.31)	0.049
**Economic Activity**						
**Yes**			–	–	–	–
**No**			2.79 (1.15–6.80)	0.024	1.91 (1.02–3.59)	0.044

^a^Chronic diseases = hypertension, heart disease, diabetes, back pain, cataracts, osteoporosis, and arthritis, loss of hearing or vision

When anxiety trajectory groups were used as the outcome, the depression groups showed significant association with the anxiety trajectories in the multivariate logistic analysis (Table 5). Female sex was a significant predictor for the low-to-middle anxiety group and the high-to-low anxiety group. Not being involved in income generating activity was another predictor of the high-to-low anxiety group membership compared to the low-flat group membership (OR = 2.17, 95% CI: 1.28–3.69, *p*-value = 0.025).

**Table 5:** Multivariate Logistic Regression Analyses with Anxiety Trajectory Groups. Estimation of odds ratio (OR) and 95% confidence interval (C.I.). Low-flat anxiety as the reference group

Variables	Low-to-middle		High-to-low		High-curved	
	OR (95% CI)	P-value	OR (95% CI)	P-value	OR (95% CI)	P-value
**Depression**						
**Low-flat**	–	–	–	–	–	–
**Low-to-middle**	8.98 (5.59–14.44)	< 0.0001	2.21 (0.99–4.94)	0.054	38.01 (11.02–131.2)	< 0.0001
**Low-to-high**	5.38 (1.59–18.25)	0.220	3.43 (0.79–14.87)	0.100	0.01 (0.01–99.9)	0.991
**High-stable**	2.10 (0.64–6.89)	0.007	14.83 (8.45–26.02)	< 0.0001	23.15 (4.18–128.3)	0.0003
**Sex**						
**Male**	–	–	–	–		
**Female**	1.73 (1.09–2.76)	0.021	2.17 (1.28–3.69)	0.025		
**Economic Activity**						
**Yes**			–	–		
**No**			1.86 (1.08–3.18)	0.025		

## Discussion

In this longitudinal study, data gathered from the KHPS included 3983 Korean older adults who were thought to have a higher potential of receiving care for depression and/or anxiety symptoms within an 8 year follow-up period. To examine the tendency to suffer from these two mental health problems simultaneously, which may change over time, GBDTM was applied to identify trajectories of co-occurring depression and anxiety. Among the four groups recognized for their differing probabilities of depression, a large majority showed no depression and were generally unlikely to experience anxiety concomitantly. However, slightly more than 10% did experience depression during the follow up period, with most of these individuals showing a gradual increase in depression probability. Among individuals following this trajectory, 20% also experienced a moderate increase in anxiety risk over time. Less than 3% of the subjects were identified as having high stable depression, and more than 30% of them had comorbid anxiety. Regarding anxiety, also recognized to follow four trajectories, the vast majority of respondents did not experience this and were also free of depression, although 5% again saw a slow increase in anxiety propensity over time. This was accompanied by an increasing depression tendency in just under half the cases. About 2% of the individuals showed a slow decrease of anxiety trend over time, and 30% of them had a high probability of depression. Only 13 (0.6%) patients were found in the trajectory showing a high curved anxiety probability, and more than half also had increasing depression over time. In general, female sex, not involved in income-generating activity, and membership in a trajectory suggesting a risk for the alternate condition independent predicted a more vulnerable risk trajectory than “low-flat” for both depression and anxiety.

Our presentation of four trajectory groups aligns with other depression trajectories studies focused on older adults [40, 41, 44, 61]. However, researchers have only infrequently identified older adult anxiety trajectories: a six-year cohort study of depression and anxiety trajectories in older adults by Holmes et al., which only involved two trajectories (a stable anxiety trajectory (82%) and an elevated anxiety trajectory (18%)) [45] and two studies recognizing three anxiety trajectory groups in older adults [46, 47]. In our study, four anxiety trajectory groups were identified. The anxiety trajectory shapes of these latter studies are different than our study as one focused on depressed older patients and the other on musculoskeletal pain patients.

Among our four depression trajectory groups, no decreasing trajectory was found for depression. One low depression trajectory group, one high depression trajectory group, and two increasing trajectories were identified in our data, consistent with findings in other studies [39–42, 44, 61]. The high-stable depression group is thought to have less likelihood of recovery in our older population as this life stage is more likely to include reduced life satisfaction, low income and living quality, and more adverse health conditions [62–64]. The low-to-high depression group had an intense increase in depression occurrence from 2009 to 2013, but this group only contained 31 older adults. The markedly increased probability may have been precipitated by sudden serious events, such as the loss of a spouse, physical incapacity, etc. However, among anxiety trajectories, a declining trajectory and a curved shape trajectory showed evidence of a decreasing risk. The declining trajectories were also identified in other studies [45, 47]. A possible explanation for this observed decline is that individuals adapt or cope better with their anxiety and no longer seek treatment. Another explanation may be that other more pressing medical conditions emerge, eclipsing anxiety management; as such anxiety may still have been present but not identified [65]. The high-curve anxiety trajectory only involved 13 older adults, which is extremely small. However, because of the constant high anxiety probability of these subjects, this group is important and cannot be replaced by other trajectory groups.

The association between depression and anxiety was clearly identified from the conditional probabilities of the trajectories and the logistic regression odds ratios. The current finding that the low-to-high and low-to-middle depression group also were at risk of being in the low-to-middle anxiety group suggests that older adults with an increasing trend of depression over time also have a greater chance of increasing anxiety, consistent with other research [66, 67]. Moreover, low-to-middle depression group members made up a high proportion in the high-curved anxiety group, suggesting that older patients who had severe anxiety may suffer mild depression as well. High-stable depression group members were more likely to have anxiety risk following the high-to-low and, less frequently, the high-curved anxiety trajectory; individuals in this particular overlap have serious mental health conditions and require more attention [2]. The association between depression and anxiety status was also supported by the inverse of these findings; individuals in this study who did not have one of the study conditions tended not to have the other either.

Our evaluation of demographic risk factors showed variable consistency with the literature. In the majority of the depression and anxiety studies, sex does have an association with these conditions, suggesting older females are generally at greater risk [68, 69]. Our study’s findings in this regard are consistent with results from other trajectory studies [43–45, 70]. Nevertheless, other studies have found no sex-specific differences when investigating depression and anxiety [47, 71]. This inconsistency may be related to different economic circumstances, social-cultural factors, psychosocial gender roles, or other population differences. In our study, age was found to be a significant univariate predictor of depression only, consistent with Holmes et al., 2018 [45]. Level of education was not a significant predictor of either outcome, which is consistent with some studies [45, 72, 73], but not others [39–44, 47, 74]. In our study, this lack of relationship may be attributable to the relatively low education level in our respondents overall.

Social factors are also known to influence mental health. Some studies suggest that older adults living alone or those having no partner within an isolated social environment have a higher risk of depression or anxiety [70, 75–79]. However, living alone and marital status were not related to the outcomes in our study, which is consistent with other studies [41–43, 46, 80]. The risk behaviors of smoking or excessive drinking might also increase the risk of depression and anxiety [40, 41, 77, 81]. Nevertheless, this association was not identified in our study or in the work of others [44, 75]. Studies show that homeownership reduces the risk of depression and anxiety [75, 82], but this association did not remain significant in the multivariate analysis. Income-generating activity, however, did predict both depression and anxiety trajectory groups, suggesting that people in later life who were still working and had financial security may have better mental health. Poor mental health and ability to work may affect each other both ways, in addition, that there may be alternative explanations for working later in life (financially insecure/secure) [83–86].

Chronic diseases (hypertension, heart disease, stroke, diabetes, asthma, cancer, arthritis, osteoporosis, back pain, cataracts, loss of hearing or vision) are understandably difficult challenges that may impact mental health in older adults. In studying the relationship between depression, anxiety, and chronic disease, Clarke and Kay reviewed 159 papers and found that depression was correlated with nearly all chronic diseases [87]. However, anxiety was only associated with heart disease, stroke, and diabetes mellitus. It was further found that patients with depression and anxiety who were also diagnosed with heart disease, stroke, cancer, and arthritis were more difficult to treat [87]. In our study, the older adults who had more than three chronic diseases were more likely to develop depression. However, in the anxiety trajectory groups, chronic disease was only significant in the “high-to-low” group from the univariate analysis, but not in the multivariate analysis. Previous studies have suggested that in older adults, physical illness or disability is generally positively correlated with depression and anxiety [75, 88–90], but in our univariate analysis, physical/mental disability was not observed to predict these outcomes.

Several limitations should be considered in this study. Firstly, in this study we moved the additional participants in 2014 to baseline measurement 2008 (Fig. S1). Since they joined the study in 2014 (6 years after the initial study time), so the social and economic situation might be changed. However, we assume the change would be ignorable. Secondly, these outcomes were collected from medical expenditures and prescription drug receipts or from medical institutions/pharmacies, potentially leading to inadequate recognition of outcomes in our sample. This is particularly true in the context of other comorbid chronic disease conditions [91]. Another limitation was the low prevalence of anxiety across the survey period which limited predictor evaluation, particularly in the poorly populated trajectories such as the “high-curved” group (*n* = 13). Furthermore, although the current study employed data from a large older adults subsample of the KHPS dataset, around 35% of the outcome measurements were missing, which might have resulted in bias even though imputations were used under the missing at random assumption. Our sensitivity analysis with complete data showed that depression had three trajectory groups (high-stable, low-to-middle, low-flat). However, anxiety had four trajectory groups, which was close to the result with imputation (Fig. S2 in Supplement Material). Another limitation is that the variables included in this study did not contain all the potentially important health and psychosocial aspects that might be associated with depression and anxiety, such as stressful life events and social/family support information. Lastly, these data were all collected from the specific cultural context of Korean older adults, which may not be generalizable to all contexts.

In summary, four trajectory groups of both depression and anxiety were generated among the older adults of the KHPS dataset. Most older adults belonged to the low-flat trajectory group for both depression (87.0%) and anxiety (92.5%), which suggests that older adults do not identify depression and anxiety as problems. However, among those who do, an interrelationship between these diagnoses, particularly in those with anxiety, was evident from GBDTM. Female sex, the presence of 3 or more chronic diseases, and involvement in income-generating activity were found to be additional predictors for a concerning depression trajectory group, and except for chronic diseases, for the anxiety trajectories as well. The findings of this study can be used to assist health policy decision-makers in identifying individuals at risk for comorbid depression and anxiety and aid in devising supports for older individuals at risk of deteriorating mental health. In this study, we applied the dual trajectory modeling method, which has been rarely used in latent class analysis. Utilizing this kind of joint modeling linking two repeated measurement outcomes with non-ignorable inter-correlation will provide a more comprehensive approach research that allows us to better understand the study group characteristics and their direction of change over time.

## Supplementary Information

**Additional file 1: Table S1**. Univariate Logistic Regression Analyses with Depression Trajectory Groups. Estimation of odds ratio (OR) and 95% confidence interval (C.I.). Low-flat depression as the reference group. **Table S2**. Univariate Logistic Regression Analyses with Anxiety Trajectory Groups. Estimation of odds ratio (OR) and 95% confidence interval (C.I.). Low-flat anxiety as the reference group. **Fig. S1**. Study flow chart. **Fig. S2**. Depression and Anxiety Trajectories with complete data (*n* = 1785). The solid line indicates the observed value; the dot line the dashed line indicates the predicted value. A and B are depression and anxiety trajectories in group-based dual trajectory modeling, respectively.

## Data Availability

Korea Health Panel Study (KHPS) data is available at https://www.khp.re.kr:444/eng/main.do

## References

[1] Byrne GJ, Pachana NA (2010). Anxiety and depression in the elderly: do we know any more?. Curr Opin Psychiatry.

[2] Lenze EJ (2003). Comorbidity of depression and anxiety in the elderly. Curr Psychiatry Rep.

[3] WPA (2017). World Population Aging. Electronic format.

[4] Isabella S (2017). South Korea is aging faster than any other developed country. QUARTZ.

[5] DeJean D, Giacomini M, Vanstone M, Brundisini F (2013). Patient experiences of depression and anxiety with chronic disease: a systematic review and qualitative meta-synthesis. Ont Health Technol Assess Ser.

[6] Alexopoulos GS (2006). The vascular depression hypothesis: 10 years later. Biol Psychiatry.

[7] Fattouh N, Hallit S, Salameh P, Choueiry G, Kazour F, Hallit R (2019). Prevalence and factors affecting the level of depression, anxiety, and stress in hospitalized patients with a chronic disease. Perspect Psychiatr Care.

[8] Flint AJ (1994). Epidemiology and comorbidity of anxiety disorders in the elderly. Am J Psychiatry.

[9] Beekman AT, De Beurs E, Van Balkom AJ, Deeg DJ, Van Dyck R, Van Tilburg W (2000). Anxiety and depression in later life: co-occurrence and communality of risk factors. Am J Psychiatr.

[10] Gould CE, O'Hara R, Goldstein MK, Beaudreau SA (2016). Multimorbidity is associated with anxiety in older adults in the health and retirement study. Int J Geriatr Psychiatry.

[11] Birk JL, Kronish IM, Moise N, Falzon L, Yoon S, Davidson KW (2019). Depression and multimorbidity: considering temporal characteristics of the associations between depression and multiple chronic diseases. Health Psychol.

[12] Read JR, Sharpe L, Modini M, Dear BF (2017). Multimorbidity and depression: a systematic review and meta-analysis. J Affect Disord.

[13] Kang HJ, Bae KY, Kim SW, Shin HY, Shin IS, Yoon JS, Kim JM (2017). Impact of anxiety and depression on physical health condition and disability in an elderly Korean population. Psychiatry Investig.

[14] Flint AJ, Rifat SL (1997). Two-year outcome of elderly patients with anxious depression. Psychiatry Res.

[15] Zhu C, Ou L, Geng Q, Zhang M, Ye R, Chen J, Jiang W (2012). Association of somatic symptoms with depression and anxiety in clinical patients of general hospitals in Guangzhou, China. Gen Hosp Psychiatry.

[16] Lenze EJ, Mulsant BH, Shear MK, Schulberg HC, Dew MA, Begley AE, Pollock BG, Reynolds CF (2000). Comorbid anxiety disorders in depressed elderly patients. Am J Psychiatr.

[17] Norton PJ, Temple SR, Pettit JW (2008). Suicidal ideation and anxiety disorders: elevated risk or artifact of comorbid depression?. J Behav Ther Exp Psychiatry.

[18] Vink D, Aartsen MJ, Schoevers RA (2008). Risk factors for anxiety and depression in the elderly: a review. J Affect Disord.

[19] Weisenbach SL, Boore LA, Kales HC (2012). Depression and cognitive impairment in older adults. Curr Psychiatry Rep.

[20] Zuckerman H, Pan Z, Park C, Brietzke E, Musial N, Shariq AS, Iacobucci M, Yim SJ, Lui LM, Rong C, McIntyre RS (2018). Recognition and treatment of cognitive dysfunction in major depressive disorder. Front Psychiatry.

[21] Yen YC, Rebok GW, Gallo JJ, Jones RN, Tennstedt SL (2011). Depressive symptoms impair everyday problem-solving ability through cognitive abilities in late life. Am J Geriatr Psychiatry.

[22] Mantella RC, Butters MA, Dew MA, Mulsant BH, Begley AE, Tracey B, Shear MK, Reynolds CF, Lenze EJ (2007). Cognitive impairment in late-life generalized anxiety disorder. Am J Geriatr Psychiatry.

[23] Butters MA, Bhalla RK, Andreescu C, Wetherell JL, Mantella R, Begley AE, Lenze EJ (2011). Changes in neuropsychological functioning following treatment for late-life generalised anxiety disorder. Br J Psychiatry.

[24] De Beurs E, Beekman A, Geerlings S, Deeg D, Van Dyck R, Van Tilburg W (2001). On becoming depressed or anxious in late life: similar vulnerability factors but different effects of stressful life events. Br J Psychiatry.

[25] Schoevers RA, Beekman AT, Deeg DJ, Jonker C, Tilburg WV (2003). Comorbidity and risk-patterns of depression, generalised anxiety disorder and mixed anxiety-depression in later life: results from the AMSTEL study. Int J Geriatr Psychiatry.

[26] Jang Y, Haley WE, Small BJ, Mortimer JA (2002). The role of mastery and social resources in the associations between disability and depression in later life. Gerontologist.

[27] Hybels CF, Blazer DG, Pieper CF (2001). Toward a threshold for subthreshold depression: an analysis of correlates of depression by severity of symptoms using data from an elderly community sample. Gerontologist.

[28] Minicuci N, Maggi S, Pavan M, Enzi G, Crepaldi G (2002). Prevalence rate and correlates of depressive symptoms in older individuals: the Veneto study. J Gerontol Ser A Biol Med Sci.

[29] Pirlich M, Schütz T, Kemps M, Luhman N, Minko N, Lübke HJ, Rossnagel K, Willich SN, Lochs H (2005). Social risk factors for hospital malnutrition. Nutrition.

[30] Acierno R, Brady K, Gray M, Kilpatrick DG, Resnick H, Best CL (2002). Psychopathology following interpersonal violence: a comparison of risk factors in older and younger adults. J Clin Geropsychol.

[31] Forsell Y (2000). Predictors for depression, anxiety and psychotic symptoms in a very elderly population: data from a 3-year follow-up study. Soc Psychiatry Psychiatr Epidemiol.

[32] Schoevers RA, Deeg DJ, Van Tilburg W, Beekman AT (2005). Depression and generalized anxiety disorder: co-occurrence and longitudinal patterns in elderly patients. Am J Geriatr Psychiatry.

[33] Heun R, Papassotiropoulos A, Ptok U (2000). Subthreshold depressive and anxiety disorders in the elderly. Eur Psychiatry.

[34] Russo J, Vitaliano PP, Brewer DD, Katon W, Becker J (1995). Psychiatric disorders in spouse caregivers of care recipients with Alzheimer's disease and matched controls: a diathesis-stress model of psychopathology. J Abnorm Psychol.

[35] Côté SM, Boivin M, Liu X, Nagin DS, Zoccolillo M, Tremblay RE (2009). Depression and anxiety symptoms: onset, developmental course and risk factors during early childhood. J Child Psychol Psychiatry.

[36] Feng X, Shaw DS, Silk JS (2008). Developmental trajectories of anxiety symptoms among boys across early and middle childhood. J Abnorm Psychol.

[37] McLaughlin KA, King K (2015). Developmental trajectories of anxiety and depression in early adolescence. J Abnorm Child Psychol.

[38] Olino TM, Klein DN, Lewinsohn PM, Rohde P, Seeley JR (2010). Latent trajectory classes of depressive and anxiety disorders from adolescence to adulthood: descriptions of classes and associations with risk factors. Compr Psychiatry.

[39] Liang J, Xu X, Quiñones AR, Bennett JM, Ye W (2011). Multiple trajectories of depressive symptoms in middle and late life: racial/ethnic variations. Psychol Aging.

[40] Kuo SY, Lin KM, Chen CY, Chuang YL, Chen WJ (2011). Depression trajectories and obesity among the elderly in Taiwan. Psychol Med.

[41] Byers AL, Vittinghoff E, Lui LY, Hoang T, Blazer DG, Covinsky KE, Ensrud KE, Cauley JA, Hillier TA, Fredman L, Yaffe K (2012). Twenty-year depressive trajectories among older women. Arch Gen Psychiatry.

[42] Hsu HC (2012). Group-based trajectories of depressive symptoms and the predictors in the older population. Int J Geriatr Psychiatry.

[43] Montagnier D, Dartigues JF, Rouillon F, Pérès K, Falissard B, Onen F (2014). Ageing and trajectories of depressive symptoms in community-dwelling men and women. Int J Geriatr Psychiatry.

[44] Kuchibhatla MN, Fillenbaum GG, Hybels CF, Blazer DG (2012). Trajectory classes of depressive symptoms in a community sample of older adults. Acta Psychiatr Scand.

[45] Holmes SE, Esterlis I, Mazure CM, Lim YY, Ames D, Rainey-Smith S, Fowler C, Ellis K, Martins RN, Salvado O, Doré V (2018). Trajectories of depressive and anxiety symptoms in older adults: a 6-year prospective cohort study. Int J Geriatr Psychiatry.

[46] Rzewuska M, Mallen CD, Strauss VY, Belcher J, Peat G (2015). One-year trajectories of depression and anxiety symptoms in older patients presenting in general practice with musculoskeletal pain: a latent class growth analysis. J Psychosom Res.

[47] Spinhoven P, van der Veen DC, Voshaar RO, Comijs HC (2017). Worry and cognitive control predict course trajectories of anxiety in older adults with late-life depression. Eur Psychiatry.

[48] Lim HJ, Cheng Y, Kabir R, Thorpe L (2020). Trajectories of depression and their predictors in a population-based study of Korean older adults. Int J Aging Hum Dev.

[49] KHPS (2016). Korea Health Panel Study.

[50] Nagin DS (1999). Analyzing developmental trajectories: a semiparametric, group-based approach. Psychol Methods.

[51] Huang DY, Lanza HI, Anglin MD (2013). Association between adolescent substance use and obesity in young adulthood: a group-based dual trajectory analysis. Addict Behav.

[52] Nagin DS, Tremblay RE (2001). Analyzing developmental trajectories of distinct but related behaviors: a group-based method. Psychol Methods.

[53] Nagin D (2005). Group-based modeling of development Harvard University press.

[54] Kessler RC, McGonagle KA, Zhao S, Nelson CB, Hughes M, Eshleman S, Wittchen HU, Kendler KS (1994). Lifetime and 12-month prevalence of DSM-III-R psychiatric disorders in the United States: results from the National Comorbidity Survey. Arch Gen Psychiatry.

[55] Caspi A, Roberts BW (2001). Personality development across the life course: the argument for change and continuity. Psychol Inq.

[56] Wiesner M, Kim HK (2006). Co-occurring delinquency and depressive symptoms of adolescent boys and girls: a dual trajectory modeling approach. Dev Psychol.

[57] Schwarz GJ (1978). Estimating the dimension of a model. Ann Statist.

[58] Akaıke H (1974). A new look at the statistical model identification. IEEE Trans Automatic Control.

[59] Kass RE, Wasserman L (1995). A reference Bayesian test for nested hypotheses and its relationship to the Schwarz criterion. J Am Stat Assoc.

[60] Nagin DS, Odgers CL (2010). Group-based trajectory modeling in clinical research. Annu Rev Clin Psychol.

[61] Xiang X, Cheng J (2019). Trajectories of major depression in middle-aged and older adults: a population-based study. Int J Geriatr Psychiatry.

[62] Dew MA, Whyte EM, Lenze EJ, Houck PR, Mulsant BH, Pollock BG, Stack JA, Bensasi S, Reynolds MDCF (2007). Recovery from major depression in older adults receiving augmentation of antidepressant pharmacotherapy. Am J Psychiatr.

[63] You KS, Lee HO, Fitzpatrick JJ, Kim S, Marui E, Lee JS, Cook P (2009). Spirituality, depression, living alone, and perceived health among Korean older adults in the community. Arch Psychiatr Nurs.

[64] Jang Y, Small BJ, Haley WE (2001). Cross-cultural comparability of the geriatric depression scale: comparison between older Koreans and older Americans. Aging Ment Health.

[65] AAPG (2019). Anxiety and Older Adults: Overcoming Worry and Fear.

[66] Wetherell JL, Gatz M, Craske MG (2003). Treatment of generalized anxiety disorder in older adults. J Consult Clin Psychol.

[67] Bassil N, Ghandour A, Grossberg GT (2011). How anxiety presents differently in older adults. Curr Psychiatr Ther.

[68] McLean CP, Asnaani A, Litz BT, Hofmann SG (2011). Gender differences in anxiety disorders: prevalence, course of illness, comorbidity and burden of illness. J Psychiatr Res.

[69] Girgus JS, Yang K, Ferri CV (2017). The gender difference in depression: are elderly women at greater risk for depression than elderly men?. Geriatrics.

[70] El-Gilany AH, Elkhawaga GO, Sarraf BB (2018). Depression and its associated factors among elderly: a community-based study in Egypt. Arch Gerontol Geriatr.

[71] Taylor MG, Lynch SM (2004). Trajectories of impairment, social support, and depressive symptoms in later life. J Gerontol Ser B Psychol Sci Soc Sci.

[72] Norris FH, Murrell SA (1988). Prior experience as a moderator of disaster impact on anxiety symptoms in older adults. Am J Community Psychol.

[73] Hong SI, Hasche L, Bowland S (2009). Structural relationships between social activities and longitudinal trajectories of depression among older adults. Gerontologist.

[74] Andreescu C, Chang CC, Mulsant BH, Ganguli M (2008). Twelve-year depressive symptom trajectories and their predictors in a community sample of older adults. Int Psychogeriatr.

[75] Kang HJ, Bae KY, Kim SW, Shin IS, Yoon JS, Kim JM (2016). Anxiety symptoms in Korean elderly individuals: a two-year longitudinal community study. Int Psychogeriatr.

[76] Chong MY, Chen CC, Tsang HY, Yeh TL, Chen CS, Lee YH, Tang TC, Lo HY (2001). Community study of depression in old age in Taiwan: prevalence, life events and socio-demographic correlates. Br J Psychiatry.

[77] Mehta KM, Simonsick EM, Penninx BW, Schulz R, Rubin SM, Satterfield S, Yaffe K (2003). Prevalence and correlates of anxiety symptoms in well-functioning older adults: findings from the health aging and body composition study. J Am Geriatr Soc.

[78] Brown JW, Liang J, Krause N, Akiyama H, Sugisawa H, Fukaya T (2002). Transitions in living arrangements among elders in Japan: does health make a difference?. J Gerontol Ser B Psychol Sci Soc Sci.

[79] Won MR, Choi YJ (2013). Are Koreans prepared for the rapid increase of the single-household elderly? Life satisfaction and depression of the single-household elderly in Korea. Sci World J.

[80] Cole MA (1979). Sex and marital status differences in death anxiety. OMEGA: J Death Dying.

[81] Kirchner JE, Zubritsky C, Cody M, Coakley E, Chen H, Ware JH, Oslin DW, Sanchez HA, Durai UN, Miles KM, Llorente MD (2007). Alcohol consumption among older adults in primary care. J Gen Intern Med.

[82] Chiao C, Weng LJ, Botticello AL (2011). Social participation reduces depressive symptoms among older adults: an 18-year longitudinal analysis in Taiwan. BMC Public Health.

[83] Lin N, Dean A, Ensel WM (2013). Social support, life events, and depression.

[84] Newby JM, Moulds ML (2011). Intrusive memories of negative events in depression: is the centrality of the event important?. J Behav Ther Exp Psychiatry.

[85] Flint AJ, Rifat SL (1997). Anxious depression in elderly patients: response to antidepressant treatment. Am J Geriatr Psychiatry.

[86] Hersen M, Van Hasselt VB (1992). Behavioral assessment and treatment of anxiety in the elderly. Clin Psychol Rev.

[87] Clarke DM, Currie KC (2009). Depression, anxiety and their relationship with chronic diseases: a review of the epidemiology, risk and treatment evidence. Med J Aust.

[88] Knight BG, Nordhus IH, Satre DD (2003). Psychotherapy with older adults. Handb Psychol.

[89] Brenes GA, Penninx BW, Judd PH, Rockwell E, Sewell DD, Wetherell JL (2008). Anxiety, depression and disability across the lifespan. Aging Ment Health.

[90] Hermans H, Evenhuis HM (2013). Factors associated with depression and anxiety in older adults with intellectual disabilities: results of the healthy ageing and intellectual disabilities study. Int J Geriatr Psychiatry.

[91] Manela M, Katona C, Livingston G (1996). How common are the anxiety disorders in old age?. Int J Geriatr Psychiatry.

