# Supplementary material for: Latent class growth modeling of depression and anxiety in older adults: an 8-year follow-up of a population-based study
Source: BMC Geriatr. 2021 Oct 13;21:550. doi: 10.1186/s12877-021-02501-6 (PMC8515663; doi:10.1186/s12877-021-02501-6)

Supplement Material

Table S1: Univariate Logistic Regression Analyses with Depression Trajectory Groups. Estimation of odds ratio (OR) and 95% confidence interval (C.I.). Low-flat depression as the reference group.

| Variables | Low-to-middle | | Low-to-high | | High-stable | |
| --- | --- | --- | --- | --- | --- | --- |
|  | **OR (95% CI)** | **P-value** | **OR (95% CI)** | **P-value** | **OR (95% CI)** | **P-value** |
| Anxiety Low-flat  Low-to-middle  High-to-low  High-curved  Sex Male  Female  Age 65-69  70-74  75-79  ≥ 80  Marriage Married Single/divorce/widower  Education None  Elementary  Middle/High  University  Living couple only  alone  others  Smoking No  Previous  Current  Drinking No  < 2 days/week  2-4 days/week  almost daily  Residential Not Metro-city  Metro-city  Housing Detached  Apartment  Others  Disability No  Yes  Home ownership Own  Lease  > 3 chronic diseases^#^ No  Yes  Walking No  ≤3 days/week  >3 days/week  Medium/Intensive Physical activity No  ≤3 days/week  >3 days/week  Economic Activity Yes  No  Income percentile < 20^th^  20 – 40^th^  40 – 60^th^  60 – 80^th^  80 – 100^th^ | -  10.33 (6.46-16.51)  2.67 (1.20-5.94)  38.01 (11.01-131.2)  -  1.98 (1.45-2.70)  -  1.09 (0.79-1.50)  0.91 (0.61-1.35)  0.31 (0.16-0.63)  -  0.97 (0.72-1.30)  -  1.22 (0.83-1.78)  0.95 (0.63-1.45)  0.66 (0.33-1.34)  -  0.26 (0.04-1.90)  0.67 (0.49-0.92)  -  0.38 (0.21-0.68)  0.73 (0.53-1.02)  -  0.73 (0.54-0.98)  0.49 (0.25-0.96)  0.48 (0.25-0.93)  -  0.93 (0.70-1.25)  -  0.76 (0.48-1.20)  1.09 (0.80-1.49)  -  1.13 (0.80-1.59)  -  0.95 (0.68-1.33)  -  4.15 (1.94-8.88)  -  1.54 (0.94-2.53)  1.12 (0.76-1.66)  -  1.08 (0.67-1.74)  1.05 (0.75-1.47)  -  1.15 (0.86-1.55)  -  0.83 (0.56-1.22)  0.96 (0.63-1.46)  0.65 (0.35-1.20)  0.31 (0.12-0.76) | -  <0.0001  0.016  <0.0001  -  <0.0001  -  0.619  0.632  0.001  -  0.816  -  0.314  0.817  0.250  -  0.184  0.012  -  0.001  0.067  -  0.039  0.036  0.029  -  0.637  -  0.239  0.597  -  0.491  -  0.758  -  0.0002  -  0.085  0.561  -  0.750  0.767  -  0.344  -  0.338  0.860  0.164  0.011 | -  6.18 (1.83-20.88)  4.41 (1.03-18.96)  0.01 (0.01-99.9)  -  2.14 (0.99-4.61)  -  1.28 (0.55-2.96)  1.53 (0.61-3.83)  0.81 (0.23-2.93)  -  1.06 (0.52-2.17)  -  0.76 (0.32-1.85)  0.84 (0.33-2.15)  0.64 (0.14-3.05)  -  1.89 (0.25-14.32)  0.94 (0.46-1.92)  -  0.41 (0.10-1.74)  0.86 (0.39-1.87)  -  0.55 (0.27-1.15)  0.28 (0.04-2.08)  0.51 (0.12-2.20)  -  1.19 (0.60-2.38)  -  1.20 (0.44-3.28)  1.31 (0.61-2.81)  -  1.11 (0.48-2.57)  -  0.73 (0.30-1.77)  -  4.70 (0.64-34.45)  -  1.80 (0.55-5.93)  1.18 (0.45-3.13)  -  0.77 (0.23-2.54)  0.31 (0.10-1.04)  -  2.69 (1.11-6.54)  -  1.16 (0.50-2.66)  1.10 (0.42-2.84)  0.98 (0.28-3.41)  0.40 (0.05-3.03) | -  0.003  0.046  0.991  -  0.053  0.566  0.359  0.752  -  0.865  0.548  0.721  0.578  -  0.989  0.873  -  0.226  0.702  -  0.112  0.974  0.365  -  0.622  -  0.718  0.488  -  0.805  -  0.485  -  0.128  -  0.335  0.738  -  0.663  0.057  -  0.029  -  0.729  0.851  0.972  0.975 | -  2.28 (0.70-7.42)  18.68 (10.94-31.9)  23.15 (4.18-128.3)  -  2.28 (1.47-3.56)  -  1.28 (0.78-2.10)  1.91 (1.15-3.19)  1.03 (0.51-2.05)  -  0.99 (0.65-1.49)  -  0.78 (0.47-1.31)  1.04 (0.62-1.75)  0.32 (0.10-1.08)  -  1.10 (0.26-4.58)  0.79 (0.52-1.20)  -  0.70 (0.37-1.32)  0.41 (0.23-0.74)  -  0.63 (0.41-0.96)  0.19 (0.05-0.79)  0.46 (0.18-1.17)  -  0.97 (0.65-1.45)  -  1.24 (0.72-2.16)  1.12 (0.72-1.75)  -  1.75 (1.14-2.69)  -  1.89 (1.26-2.83)  -  7.49 (1.84-30.44)  -  0.89 (0.47-1.67)  0.53 (0.33-0.85)  -  0.65 (0.30-1.43)  0.57 (0.33-1.00)  -  2.23 (1.39-3.59)  -  0.74 (0.41-1.36)  0.56 (0.26-1.21)  0.88 (0.39-1.99)  0.14 (0.02-1.04) | -  0.172  <0.0001  <0.0001  -  0.0003  -  0.332  0.013  0.943  -  0.945  -  0.349  0.883  0.066  -  0.900  0.271  -  0.267  0.003  -  0.031  0.022  0.105  -  0.880  -  0.442  0.618  -  0.010  -  0.002  -  0.005  -  0.713  0.008  -  0.285  0.051  -  0.0009  -  0.334  0.142  0.754  0.055 |

^#^ Chronic diseases = hypertension, heart disease, diabetes, back pain, cataracts, osteoporosis, and arthritis, loss of hearing or vision.

Table S2: Univariate Logistic Regression Analyses with Anxiety Trajectory Groups. Estimation of odds ratio (OR) and 95% confidence interval (C.I.). Low-flat anxiety as the reference group.

| Variables | Low-to-middle | | High-to-low | | High-curved | |
| --- | --- | --- | --- | --- | --- | --- |
|  | **OR (95% CI)** | **P-value** | **OR (95% CI)** | **P-value** | **OR (95% CI)** | **P-value** |
| Depression Low-flat  Low-to-middle  Low-to-high  High-stable  Sex Male  Female  Age 65-69  70-74  75-79  ≥ 80  Marital status Married Single/divorce/widower    Education None  Elementary  Middle/High  University  Living arrangement Couple only  Alone  Others  Smoking No  Previous  Current  Drinking No  < 2 days/week  2-4 days/week  Almost daily  Residential Not Metro-city  Metro-city  Housing Detached  Apartment  Others  Disability No  Yes  Home ownership Own  Lease  >3 chronic diseases ^#^ No Yes    Walking No  ≤3 days/week  >3 days/week  Medium/Intensive Physical activity  No  ≤3 days/week  >3 days/week  Economic Activity Yes  No  Income quantile, percentile < 20^th^  20 – 40^th^  40 – 60^th^  60 – 80^th^  80 – 100^th^ | -  10.33 (6.46-16.5)  6.18 (1.83-20.88)  2.28 (0.70-7.42)  -  2.11 (1.34-3.33)  -  0.94 (0.58-1.52)  1.01 (0.58-1.76)  0.71 (0.34-1.48)  -  1.19 (0.78-1.80)  -  1.17 (0.69-1.98)  0.73 (0.40-1.35)  0.52 (0.18-1.54)  -  0.57 (0.08-4.19)  0.70 (0.45-1.10)  -  0.43 (0.18-0.99)  0.79 (0.49-1.27)  -  1.10 (0.71-1.71)  0.57 (0.20-1.60)  1.23 (0.59-2.57)  -  0.97 (0.64-1.47)  -  0.77 (0.40-1.47)  0.88 (0.55-1.41)  -  0.87 (0.51-1.48)  -  0.75 (0.45-1.27)  -  1.54 (0.74-3.21)  -  1.05 (0.48-2.31)  1.24 (0.71-2.19)  -  1.05 (0.52-2.15)  1.31 (0.83-2.09)  -  1.03 (0.67-1.56)  -  0.84 (0.49-1.45)  0.69 (0.35-1.34)  1.02 (0.49-2.11)  0.40 (0.12-1.29) | -  <0.0001  0.003  0.172  -  0.001  -  0.788  0.975  0.359  -  0.423  -  0.562  0.316  0.240  -  0.582  0.122  -  0.047  0.329  -  0.666  0.283  0.587  -  0.885  -  0.425  0.603  -  0.605  -  0.286  -  0.243  -  0.899  0.452  -  0.885  0.253  -  0.903  -  0.54  0.27  0.96  0.13 | -  2.67 (1.20-5.94)  4.41 (1.03-18.96)  18.68 (10.94-31.9)  -  2.71 (1.64-4.47)  -  0.88 (0.53-1.47)  0.90 (0.50-1.62)  0.83 (0.41-1.68)  -  1.54 (1.01-2.36)  -  0.68 (0.40-1.15)  0.69 (0.39-1.22)  0.55 (0.21-1.44)  -  0.01 (0.01-99.9)  1.04 (0.68-1.60)  -  0.75 (0.38-1.46)  0.48 (0.27-0.87)  -  0.72 (0.46-1.13)  0.48 (0.17-1.34)  0.34 (0.11-1.12)  -  0.91 (0.59-1.41)  -  1.40 (0.79-2.49)  1.10 (0.68-1.80)  -  1.49 (0.93-2.40)  -  1.66 (1.06-2.60)  -  6.10 (1.50-24.87)  -  0.46 (0.19-1.08)  0.70 (0.43-1.15)  -  0.88 (0.42-1.85)  0.68 (0.39-1.20)  -  2.17 (1.30-3.62)  -  1.13 (0.63-2.04)  1.15 (0.60-2.22)  0.80 (0.31-2.08)  0.38 (0.09-1.58) | -  0.016  0.046  <0.0001  -  0.0001  -  0.634  0.716  0.607  -  0.044  -  0.154  0.204  0.221  -  0.982  0.858  -  0.393  0.015  -  0.154  0.158  0.076  -  0.663  -  0.250  0.676  -  0.099  -  0.026  -  0.012  -  0.074  0.161  -  0.730  0.186  -  0.003  -  0.680  0.675  0.651  0.183 | -  38.01 (11.0-131.2)  0.01 (0.01-99.9)  23.15 (4.18-128.3)  -  1.77 (0.54-5.74)  -  1.26 (0.31-5.04)  1.55 (0.35-6.96)  1.54 (0.28-8.42)  -  0.84 (0.26-2.73)  -  2.10 (0.45-9.76)  0.33 (0.03-3.67)  1.31 (0.12-14.47)  -  0.01 (0.01-99.9)  1.05 (0.34-3.22)  -  0.01 (0.01-99.9)  1.34 (0.44-4.10)  -  0.91 (0.29-2.82)  0.87 (0.11-7.28)  0.01 (0.01-99.9)  -  1.01 (0.33-3.09)  -  0.59 (0.07-4.79)  1.43 (0.45-4.52)  -  0.01 (0.01-99.9)  -  2.80 (0.94-8.35)  -  99.9 (0.01-99.9)  -  0.96 (0.16-5.75)  0.72 (0.19-2.72)  -  0.01 (0.01-99.9)  0.52 (0.12-2.35)  -  1.33 (0.41-4.31)  -  0.20 (0.03-1.53)  0.27 (0.04-2.15)  0.01 (0.01-99.9)  0.58 (0.07-4.58) | -  <0.0001  0.991  0.0003  -  0.345  -  0.746  0.565  0.620  -  0.773  -  0.342  0.369  0.827  -  0.989  0.929  -  0.969  0.612  -  0.865  0.900  0.976  -  0.986  -  0.620  0.541  -  0.961  -  0.065  -  0.970  -  0.961  0.628  -  0.973  0.396  -  0.640  -  0.119  0.218  0.973  0.607 |

Figure S1: Study flow chart


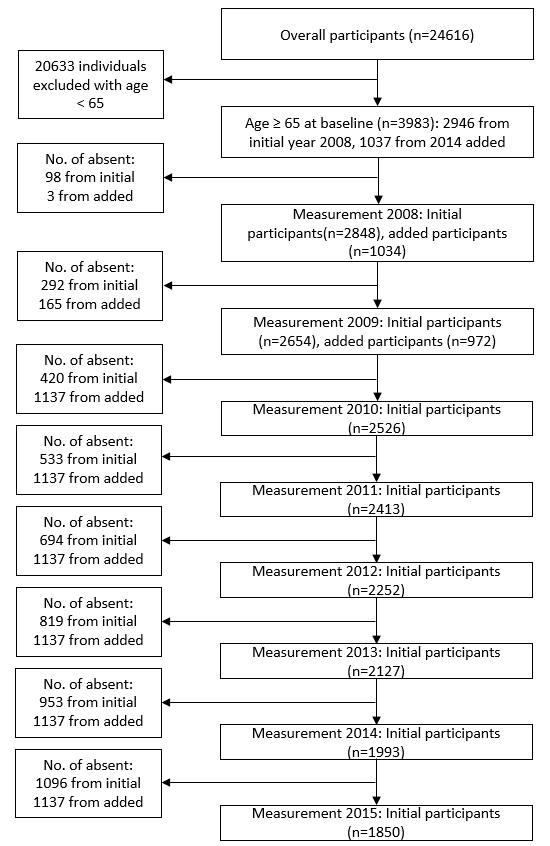


Figure S2: Depression and Anxiety Trajectories with complete data (n=1,785). The solid line indicates the observed value; the dot line the dashed line indicates the predicted value. A and B are depression and anxiety trajectories in group-based dual trajectory modeling, respectively.

1. B.


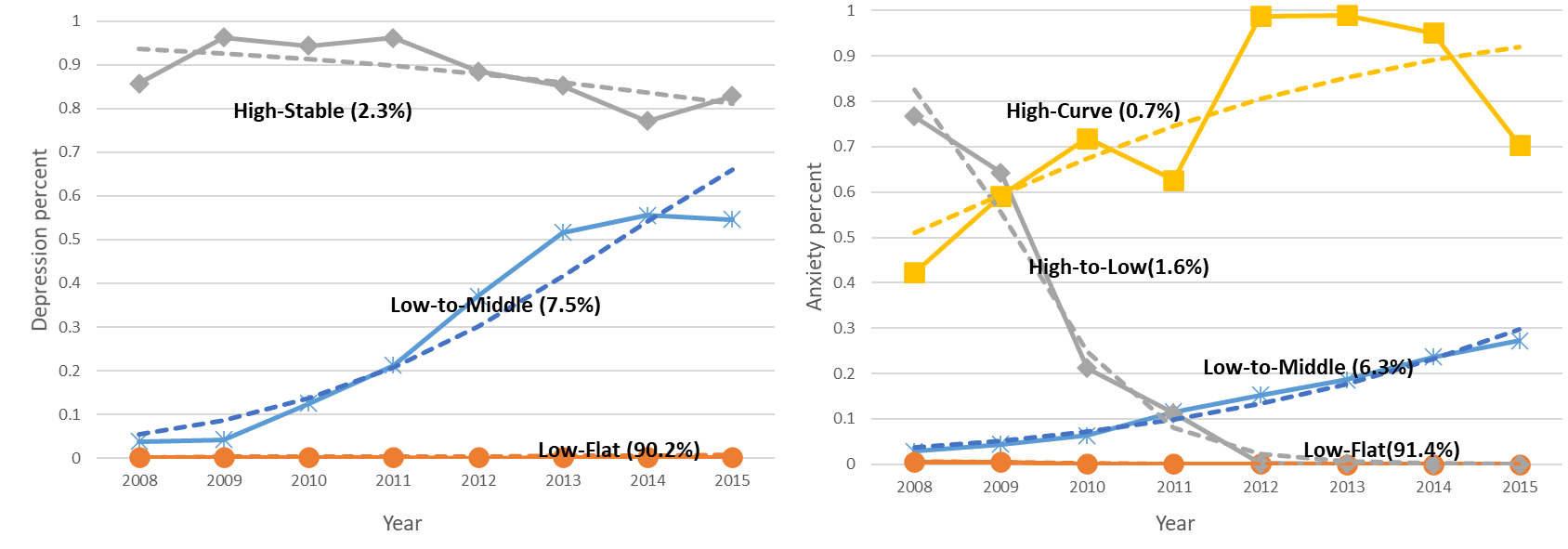

Supplement: Supplementary file 1 — Additional file 1: Table S1. Univariate Logistic Regression Analyses with Depression Trajectory Groups. Estimation of odds ratio (OR) and 95% confidence interval (C.I.). Low-flat depression as the reference group. Table S2. Univariate Logistic Regression Analyses with Anxiety Trajectory Groups. Estimation of odds ratio (OR) and 95% confidence interval (C.I.). Low-flat anxiety as the reference group. Fig. S1. Study flow chart. Fig. S2. Depression and Anxiety Trajectories with complete data (n = 1785). The solid line indicates the observed value; the dot line the dashed line indicates the predicted value. A and B are depression and anxiety trajectories in group-based dual trajectory modeling, respectively. [file 12877_2021_2501_MOESM1_ESM.docx]
